# Plastic waste interferes with chemical communication in aquatic ecosystems

**DOI:** 10.1038/s41598-019-41677-1

**Published:** 2019-04-10

**Authors:** B. Trotter, A. F. R. M. Ramsperger, P. Raab, J. Haberstroh, C. Laforsch

**Affiliations:** Department of Animal Ecology I and BayCEER, University of Bayreuth, Bayreuth, Germany

## Abstract

Environmental pollution with plastic waste has gained increasing attention, as the contamination of aquatic habitats poses a challenge to these ecosystems. Plastic waste has direct negative effects on animals such as reduced growth rate, fecundity or life span. However, the indirect effects of plastic waste, which has the ability to sorb chemicals from the surrounding media, on chemical communication have yet to be investigated. Chemical communication is crucial for aquatic organisms, e.g., to avoid predation. The planktonic water flea *Daphnia* (Crustacea), an important link between trophic levels, relies on info-chemicals (kairomones) to assess its current predation risk and to form inducible defences. We show that plastic waste, composed of high-density polyethylene (HDPE) and polyethylene terephthalate (PET) interferes with the formation of inducible defences in *Daphnia longicephala* when exposed to a combination of kairomones of *Notonecta glauca* and plastic waste. *D*. *longicephala* shows a reduction in all defensive traits, including body length, crest width and time until primiparity, compared to exposure to solely kairomone conditioned media. Plastic waste in the absence of kairomones had no effect on defensive traits. Since it is vital to adjust these defences to the current predation risk, any misperception can have far-reaching ecological consequences. Therefore, plastic waste can have indirect effects on organisms, which may manifest at the community level.

## Introduction

Humans have substantially influenced and modified ecosystems in the past by introducing anthropogenic pollutants and waste into these systems. As plastic makes up the majority of anthropogenic waste, its presence is a topic of interest. Since the introduction of petroleum-based polymers in the early 20^th^ century, the production of plastic has risen to 335 Mtons in 2016^1^. The great amount of plastic produced is accompanied by a huge amount of waste, as many products are produced for only short-term use, e.g., packaging materials, of which 32% are discarded inappropriately^2^. With this trend steadily continuing, the introduction of plastic waste into the environment will increase as well, affecting not only marine but also limnetic systems^3^. The introduction of plastics into the environment poses a great challenge to these ecosystems, as it may cause detrimental effects on biota. The consequences for organisms are still being evaluated, but several properties of plastics render them problematic. Both macro-(>5 mm) and microplastic particles (<5 mm) can be taken up by a wide variety of taxa^4^. This uptake can have a multitude of direct effects on organisms, such as reduced growth rate or starvation^5,6^. Furthermore, plastics not only leach chemicals out of their matrices but also adsorb and accumulate a multitude of organic substances onto their surfaces, thus forming a so-called eco-corona^7,8^. This condition leads to the question of whether semiochemicals, used in chemical communication among aquatic organisms, can adsorb to plastics. Chemical communication plays an important role in aquatic environments. A variety of taxa respond to minute concentrations of chemical cues released by other organisms, for example, to avoid predation or find mating partners^9^. Among the most well-studied examples is the response of prey to so-called kairomones, semiochemicals emitted by a predator and perceived by its prey, allowing the prey to assess the current predation risk and to form inducible defences^10^.

Predator-induced formations of protective traits in the crustacean *Daphnia* (water flea) serve as a textbook example of this phenotypic plasticity and comprise behavioural, life-history and morphological changes^11^. *D*. *longicephala*, for instance, responds to the kairomones released by the insect predator *N*. *glauca* with the formation of a distinct dorsal crest, a larger body size at primiparity (carrying of the first brood in the brood pouch) and delayed maturity^12^ (see supplementary material Fig. S1 for comparison of induced (predator exposed) and non-induced (control) *D*. *longicephala*).

It is well documented that chemical anthropogenic pollution, such as pharmaceuticals can interfere with the chemical communication between aquatic organisms^13,14^. Further, in *Daphnia* it has been shown that contaminants such as copper are interfering directly with the olfactory system, thus reducing the prey’s ability to detect the kairomone^15^. However, it is not clear whether particulate anthropogenic litter, such as plastic waste, interferes with chemical communication as well by adsorbing chemical substances to its surface. Hence, the goal of our study was to test whether kairomones adsorb to plastics, thus reducing the perceivable kairomone concentration in the environment. This condition would result in the reduced formation of inducible defences as the process of the formation of these phenotypically plastic defences follows a dose response curve, as shown in studies by Tollrian *et al*.^10^, Laforsch *et al*.^16^ and Elert and Pohnert^17^.

## Results

**Figure 1:**
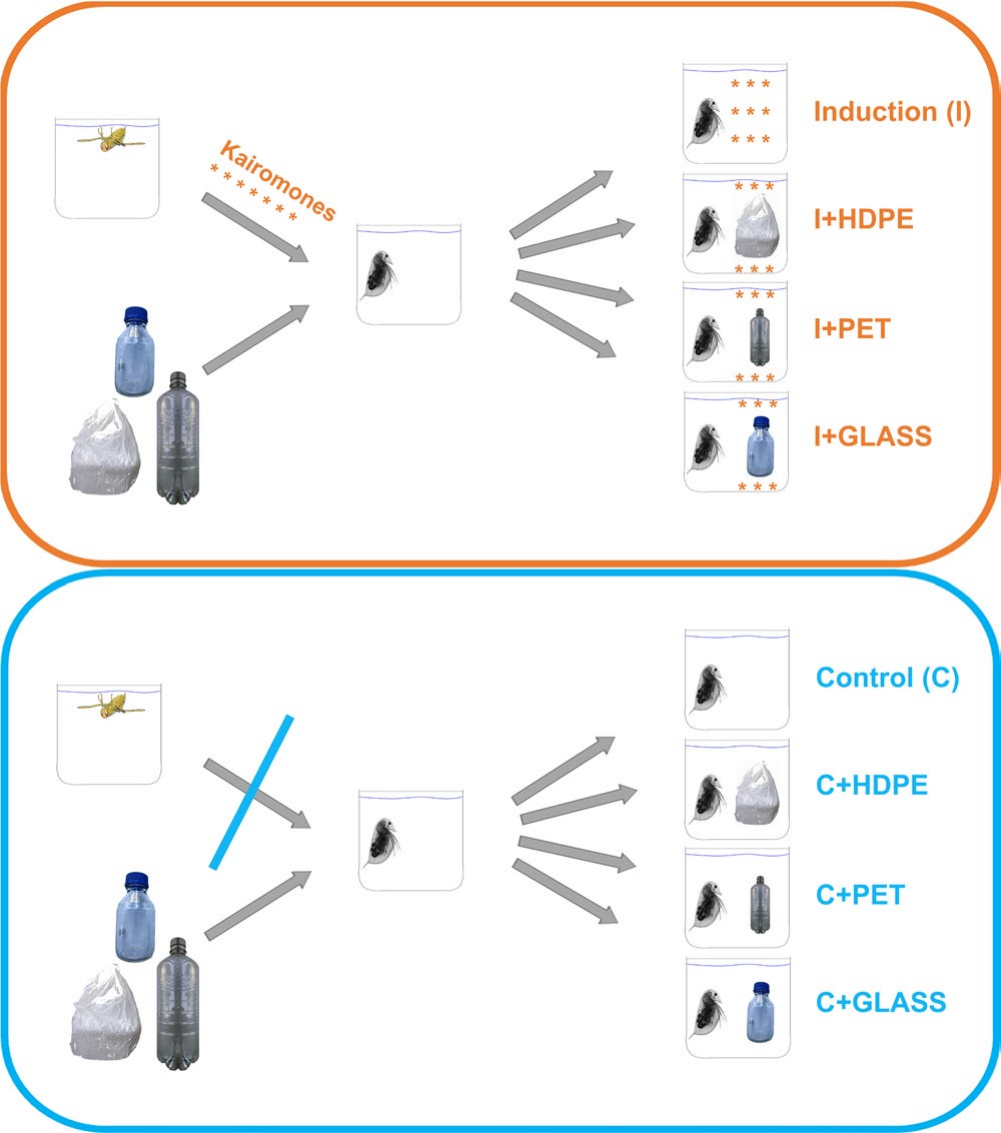
Schematically drawing of experimental setup: Orange encircled treatments represent the kairomone (from notonectid predators – top left) exposed daphnids. (I), plastic-free, solely kairomone-containing; (I + HDPE; I + PET kairomone and the respective plastic. (I + GLASS) positive surface control to the plastic treatments. Orange asterisks indicate kairomones. Blue encircled treatments represent the daphnids exposed to kairomone free medium. (C), plastic- and kairomone free, (C + HDPE, C + PET) only plastic, in order to exclude effects caused by plastic; (C + GLASS) only glass, in order to exclude effects caused by glass.

Here, we show that plastic waste influences predator-prey interactions in aquatic ecosystems by interfering with interspecific chemical communication. *D*. *longicephala* were exposed to a single piece of high-density polyethylene (HDPE) or polyethylene terephthalate (PET) as macroplastic waste (both with a surface area of 20.64 cm², see materials and methods section for detailed information) in combination with kairomones released by *Notonecta glauca* (I + HDPE and I + PET; see Fig. 1 for schematic experimental setup). A plastic-free, solely kairomone-containing treatment (I) served as a reference. Further controls were: i, one treatment lacking kairomones and plastics (C); ii, one treatment with *D*. *longicephala* being solely exposed to HDPE (C + HDPE) or iii; PET (C + PET) (see Fig. 1 for schematic experimental setup). Furthermore, treatments using glass slides with the same surface area as the used plastic pieces (I + GLASS; C + GLASS) were implemented as a surface control, since glass is regarded as inert^18^. The daphnids’ body length (BL), absolute crest width (CW) and time until primiparity were recorded (see supplementary material Fig. S2 for explanation).

Solely kairomone-exposed *D*. *longicephala* (I) showed a significantly larger body length (BL) (one-way ANOVA, F_7,245_ = 177.636; p < 0.001) (Tukey-HSD; p < 0.001), crest width (CW) (Welch F Test, F_7,103.358_ = 1149.282; p < 0.001) (Tamhane T2; p < 0.001) and delayed-maturity (Kruskal-Wallis test, χ^2^ (7) = 138.367; p < 0.001) compared to the control animals (C) (Tables 1 and 2) thus showing successful induction of morphological traits (see supplementary material Fig. S3).

**Table 1:** Parameters at primiparity and number of measured *D*. *longicephala* exposed to kairomones.

	Induction	I + GLASS	I + HDPE	I + PET
Body length [µm]	2810.13 ± 20.86	2668.28 ± 18.28	2699.36 ± 18.13	2677.19 ± 20.51
Absolute crest width [µm]	1795.49 ± 19.04	1620.69 ± 16.42	1696.27 ± 13.30	1605.79 ± 22.70
Days until primiparity	13.48 ± 0.20	12.26 ± 0.19	12.19 ± 0.19	12.22 ± 0.18
n	30	30	32	31

Body length (BL), absolute crest width (CW) and days until primiparity of kairomone-exposed *D*. *longicephala*. n stating the number of replicates. Data show the means ± 1 SE.

**Table 2:** Parameters at primiparity and number of measured *D*. *longicephala* in the kairomone-free treatment.

	Control	C + GLASS	C + HDPE	C + PET
Body length [µm]	2241.29 ± 17.84	2254.42 ± 18.00	2280.95 ± 18.79	2221.49 ± 18.52
Absolute crest width [µm]	815.28 ± 8.86	823.75 ± 7.67	846.71 ± 9.15	811.85 ± 8.56
Days until primiparity	9.88 ± 0.21	10.09 ± 0.23	10.15 ± 0.2	10.50 ± 0.29
n	33	34	33	30

Body length (BL), absolute crest width (CW) and days until primiparity of kairomone-exposed *D*. *longicephala*. n stating the number of replicates Data show the means ± 1 SE.

**Figure 2:**
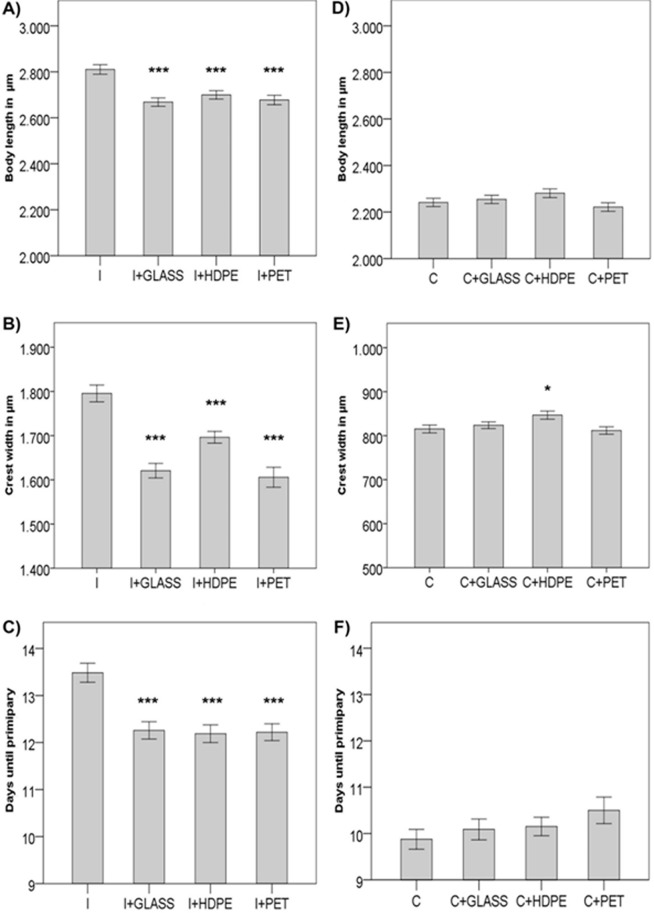
Effects of plastic waste on morphological and life history parameters of D. longicephala: (**A**) body length of daphnids exposed to kairomones; (**B**) crest width of daphnids exposed to kairomones; (**C**) duration in days until daphnids reach primiparity when exposed to kairomones. (**D**) body length of daphnids exposed to control medium; (**E**) crest width of daphnids exposed to control medium; (**F**) duration in days until daphnids reach primiparity when exposed to control medium. Error bars indicate standard error of means (±1SE). Asterisks represent level of significance when treatments are compared with induction (**I**) or control (**C**) treatments: n.s. = p ≥ 0.05; *p ≤ 0.05; ***p ≤ 0.001.

Solely kairomone-exposed daphnids (I) showed the typical significant responses to predator cues^19,20^ (Fig. 2C; see detailed results in supplementary material). However, the response of daphnids exposed to kairomones and plastic waste (I + HDPE, I + PET) simultaneously was significantly affected on a morphological and life history level compared to solely kairomone-exposed daphnids (I) by showing a reduction in body length (BL) (one-way ANOVA, F_3,119_ = 11.121; p < 0.001) (Tukey-HSD, p < 0.001) (Fig. 2A), absolute crest width (CW) (one-way ANOVA, F_3,119_ = 22.555; p < 0.001) (Fig. 2B) and days until primiparity (Kruskal-Wallis, (χ^2^ (3) = 25.894, p < 0.001) (Fig. 2C).

## Discussion

Kairomones seem to have adsorbed to the plastic waste, thus reducing the perceivable kairomone concentration. Yet, as most kairomones are still not chemically characterized^17,21–25^ (see further details in supplementary material) it was not feasible to measure kairomone concentration, neither in the water nor adsorbed to plastic. The only way to validate kairomone concentration is by the reaction norm of the daphnids, as the formation of defensive structures follows a dose-response curve^10^. The formation of smaller defensive structures makes *Daphnia* more prone to predation, as a reduction in body size, or even small structures such as neck spines, leads to a higher strike efficiency by the predator^26^. Mirza and Pyle^27^ found that neonates of *D*. *pulex* had a greater survival rate when they increased their body length by only 4–7% when exposed to kairomones of *Chaoborus*. In this context, crested *D*. *longicephala* also exhibited higher survival, evasion and escape rates compared to uncrested individuals^19^. The formation of the crest in *D*. *longicephala* is dependent on predator density, indicated by kairomone concentration, and increases in size once more predators are present^19^. Further, kairomone exposure leads to a trade-off between larger body size and time when primiparity is reached, resulting in a shift to delayed maturation^28^. In our experiment, solely kairomone-induced *D*. *longicephala* (I) took longer to reach primiparity compared to those in the plastic and kairomone treatments (I + HDPE, I + PET). These findings indicate that *D*. *longicephala* exposed to plastics and kairomones (I + HDPE, I + PET) adjusts its defensive traits to an inaccurate predator density, which renders them more vulnerable to predation^10^.

The adsorbtion of kairomones to particulate material is buttressed by the results obtained in the I + GLASS treatment, therefore diminishing an alternative hypothesis, that volatile compounds of the plastics interfere with the olfactory system of *D*. *longicephala*, as shown with copper in the study by DeMille *et al*.^15^. As glass lacks incorporated additives no leachates should interfere with the olfactory system of *D*. *longicephala*. The observed decrease in body length, crest width and days until primiparity of I + GLASS daphnids therefore shows, that kairomones have adsorbed not only to the plastic, but also the glass slides thus showing that glass itself seems not to be inert as stated in by Watcharotone *et al*.^18^.

Exposure to plastics in kairomone-free medium had no effect on any of the investigated traits except for a slightly increased CW in HDPE-exposed daphnids (Tukey HSD, p = 0.049) (Fig. 2D–F; see detailed results in supplementary material). Leaching of additives seems unlikely due to the short exposure time of <24 h, therefore potential endocrine effect of these compounds can be ruled out (for further discussion on effects of leaching see supplementary material).

The reduction of defensive traits in *D*. *longicephala* exposed to predator kairomones and plastic likely results from adsorption of the kairomones to the plastic surface. However, whether physisorption and/or chemisorption has taken place is difficult to assess, as the exact chemical structure of kairomones is not characterized^17,21–25^.

The adsorption process of kairomones in nature is likely dependent on the formation of a so called eco-corona^7,8^, which substantially builds on the surface of the plastic particles. This eco-corona (EPS or biofilm) consists of microbes and their excreted metabolic products and also a variety of organic deposits^8,29^. Since an eco-corona forms very rapidly^30^ and exudates of different trophic levels (the algae *Acutodesmus obliquus* as an autotrophic organism, *D*. *longicephala* as a heterotrophic consumer of the first order, and *N*. *glauca*, as a heterotrophic consumer of the second order, as well as the microbial community, which is associated with those organisms) were present in our setup, our experiment basically resembles natural systems, when new plastic is introduced into the ecosystem. This is corroborated by the fact that after 24 h all experimental containers showed a visible layer of biofilm on the bottom, as well as on the inserted plastic (PET) or glass slides. Hence, even if the interaction of kairomones with natural organic macromolecules and possible interference with binding sites on the plastic surfaces cannot be directly measured, given the lack of information on their chemical nature, our results show that plastic pieces which are introduced into aquatic ecosystems can rapidly interfere with chemical communication.

Until now, studies of the effects of anthropogenic stressors on chemical inter- and intraspecific communication in aquatic ecosystems solely concentrated on soluble compounds, as reviewed by Lürling and Scheffer (2007)^31^. For instance, the western spadefoot toad, *Pelobates cultripes*, did not reduce its swimming behaviour, a typical response to water-borne chemical cues released by its predators, if humic acid or ammonium nitrate was present^14^. Demille *et al*.^15^ demonstrated that *Daphnia pulicaria* was unable to respond to kairomones of the phantom midge larvae *Chaoborus* when exposed to kairomones and copper at the same time^15^. Further, the larvae of the orange clownfish, *Amphiprion percula* detects predators by olfactory cues and therefore avoids predation by the bluespotted hind, *Cephalopholis cyanostigma* and brown dottyback, *Pseudocromis fescus*. Yet, it was shown by Dixson *et al*. (2010) that olfaction in this larvae is disrupted by alterations in pH levels of the water^32^. Thus, soluble substances can interfere with the formation of defence structures, which can be maladaptive in the presence of a predator. In contrast, the indirect effects of particulate anthropogenic litter on chemical inter- and intraspecific communication in aquatic ecosystems has rarely been studied. Besseling *et al*.^6^ investigated the effects of polystyrene (PS) particles in the nano-size range when combined with fish kairomones. They showed that while *D*. *magna* typically experienced a reduction in body length (BL) in response to fish kairomones, the reduction was enhanced when PS particles and kairomones were present. At first glance, our results seem to contradict these findings. In our experiment, the presence of plastics in the kairomone-exposed daphnids also led to a reduction in defence structures, yet there was a distinct difference: the effect of kairomones was enhanced in the Besseling *et al*. study, whereas in our study, the effect was weakened. This difference might be explained as follows: Given the high surface-to-volume ratio of the nano-sized PS particles used by Besseling *et al*., it is likely that the kairomones become highly adsorbed. However, as the particles were reared in fish kairomone-treated water and then transferred to pure medium containing no kairomones at all, particles loaded with adsorbed kairomones desorbed them after the transfer. This process resulted in a high kairomone concentration in the experimental medium, indicating that kairomones are showing the same desorption behaviour as hazardous hydrophobic organic chemicals (reviewed by Koelmanns *et al*.^33^), such as polychlorinated biphenyls PCBs (e.g. IUPAC number 28 or Dichlorodiphenyldichloroethylene (DDE))^34,35^. Further, Besseling *et al*. state that leaching of additives is negligible as glass-liquid temperature of the used particles is higher than the temperature in their experiment, making leaching of compounds and therefore a potential endocrine effect very unlikely. The often-reported reduction in growth of *Daphnia*^36,37^ when exposed to nano- or microplastics may also enhance the reduction in body size in the Besseling *et al*.^6^ study.

Tollrian *et al*.^23^ were able to bind kairomones to phenol-silica cartridges and elute them later, with the elute still being able to induce defences in *Daphnia*. These findings, combined with our results, indicate that kairomones physisorb to a multitude of different materials (in our case, PET, HDPE and GLASS) while remaining active for a certain amount of time as the activity window of kairomones is limited by bacterial degradation^21,24^.

It is currently assumed that the novel risk arising from ingested plastics is dependent on the complex interaction of the constituent synthetic polymers with the environment. The resulting effects on tissues and cells of individual organisms after ingestion may then lead to ecological consequences^8^. However, the indirect effects of plastic waste on chemical inter- and intraspecific communication in aquatic ecosystems, without particle ingestion, has yet to be considered. As kairomones seem to show a similar sorption behaviour as that of organic pollutants^34,35^, plastic debris can interfere with chemical communication, as these hydrophobic chemicals have a higher affinity for binding to plastics than to natural sediment^38^. The constant influx of plastics into freshwater environments (worldwide, it is estimated that 4 to 12 Mtons are transported by rivers each year^39^) generates an increase in surfaces to which kairomones might adsorb. A lower perceptible concentration of kairomones leads to a less distinct formation of defensive traits, as defences are adjusted to the density of both predators and conspecifics^15^. Possible consequences of this maladaptation in nature are difficult to assess. To allow the transfer of knowledge we obtained in this study, performed in a lab based environment with controlled and constant parameters, out into the environment proves difficult, as we cannot measure kairomone concentrations and environmental concentrations of plastics are still poorly understood.

However, adjusting defensive strategies to the wrong predator composition or density may have effects at the population level, affecting population dynamics in higher orders of the food web. For instance, population dynamics are strongly influenced by the absence or presence of inducible defence structures. Vershoor *et al*.^40^ showed in simplified planktonic food webs that the dynamics of the algae *Scenedesmus* fluctuated by several orders of magnitude, sometimes leading to extinction if their populations were not able to express inducible defences. This finding shows the importance of the correct formation of the prey’s defences, as these fluctuations are especially critical for the persistence of species in higher trophic levels, if they are present in low densities. Maladaptations, such as those analysed in our study, may therefore have far-reaching effects on population dynamics across multiple trophic levels, as *Daphnia* occupies a central position in limnetic food webs.

## Materials and Methods

### Animal husbandry

For the experiment, we used a laboratory-cultured clone of *Daphnia longicephala* originating from a pond in southern Australia. *Notonecta glauca* were isolated from ponds at the University and were disinfected prior to the experiment using a disinfectant (General Tonic, JBL, Germany) according to the manufacturer’s instructions. This process ensures that no parasites or epibionts are introduced into the experiment. Kairomones of the European notonectid are known to induce distinct defence structures comparable to those evoked by Australian notonectids in *D*. *longicephala*^19,20^. Both *D*. *longicephala* and *N*. *glauca* were reared in semi-artificial medium (SSS-medium) based on ultra-pure water, phosphate buffer, trace elements and tap water. *D*. *longicephala* were kept in a climate chamber at a temperature of +20 °C and a day and night rhythm of 14:9 h, with a half hour each of dusk and dawn. Twelve *D*. *longicephala* were kept in 1.5 L jars containing SSS-medium. Daphnids were fed with the unicellular algae *Acutodesmus obliquus* ad libitum 3 days per week, with a weekly 50% medium change, prior to the experiment. *N*. *glauca* were kept individually in 750 mL jars in a climate chamber with dim lighting and a temperature of +8 °C prior to the experiment and were fed with five *D*. *longicephala* and 10 *Chironomidae* larvae three times per week.

### Treatments

To test the influence of plastic waste on the formation of inducible defences in *D*. *longicephala* when exposed to *N*. *glauca* kairomone, the following materials were used to simulate plastic waste: a shopping bag made of high-density polyethylene foil (HDPE) and a food container made of polyethylene terephthalate (PET). As a positive control surface, a piece of a microscope slide made of glass (GLASS) was used. PET and GLASS were cut into pieces of 37.3 mm × 25.9 mm × 1.04 mm, amounting to a cuboid with 6 surfaces and a total area of approximately 20.64 cm² per piece. The used HDPE was a thin foil with a lower density than that of PET or GLASS and therefore was positively buoyant, causing it to float on the water surface. Therefore, HDPE pieces were cut into pieces of 45.4 mm × 45.4 mm × < 0.010 mm (not measurable with Vernier callipers) to ensure approximately the same contact area (20.61 cm² per piece) with the surrounding water. Particles did not block out light, as they were transparent. The following treatments (see Fig. 1) were established, in which *Daphnia* was exposed to kairomones and plastic waste:

1. Induction (I): Kairomone medium, containing solely kairomone as a positive control for the induction.
2. Induction + glass (I + GLASS): Kairomone medium containing the glass piece.
3. Induction + HDPE (I + HDPE): Kairomone medium containing the piece of HDPE.
4. Induction + PET (I + PET): Kairomone medium containing the piece of PET.

To exclude possible effects of the materials on *Daphnia*, the respective controls were established as used in the kairomone treatment, with the difference being that the medium was not kairomone enriched, resulting in the following treatments:

1. Control (C): Pure medium as a general reference.
2. Control + glass (C + GLASS): Pure medium containing the glass piece.
3. Control + HDPE (C + HDPE): Pure medium containing the piece of HDPE.
4. Control + PET (C + PET): Pure medium containing the piece of PET.

### Media preparation and experimental procedures

To standardize the kairomone concentration for all kairomone treatments, medium was enriched with *Notonecta* kairomones and pooled prior to the daily medium change. Since the active chemical cue is not known (see supplementary material for more information regarding this subject), this step was achieved by placing one specimen of *N*. *glauca* into a 1 L glass jar containing 750 mL of SSS-medium, a 75 mm × 26 mm piece of fly screen made out of cotton (Tesa SE, Germany) for the notonectid to rest on, and five adult *D*. *longicephala* as food. *N*. *glauca* were stored in the 20 °C climate chamber one day prior the experiment as described above. After 24 h, the kairomone-enriched medium was filtered with filter paper (Whatman filter paper; pore size: 6 µm; GE Healthcare Europe GmbH, Dassel, Germany) and pooled. The same procedure was repeated with the control medium, with the difference that *N*. *glauca* were absent in the jars. 50 mL of the pooled medium was then added to 110 mL jars containing the respective treatment and *A*. *obliquus* as food for *Daphnia*, amounting to a carbon concentration of 2 mg/L.

At the start of the experiment, age-synchronized neonate *D*. *longicephala* (less than 24 h old and thus not yet sensitive to kairomones) were randomly chosen and individually placed into a 110 mL jar filled with 50 mL of the respective medium. A total of 35 replicates per treatment were carried out. Media, plastic waste and food were changed daily by transferring *D*. *longicephala* into a new jar. This process was continued until *D*. *longicephala* reached primiparity, meaning when the first brood was visible in the brood chamber, thus marking the end of the experiment. At the end of the experiment, the number of daphnids that were analysed is shown in Tables 1 and 2. The following parameters were analysed by using a Leica M50 stereo microscope equipped with a cold light source (Leica KL 300 LED, Leica Microsystems, Germany) and a digital camera (Olympus DP26, 5 Megapixel, Olympus Corporation, Japan) in combination with the software cellSens Dimension (Olympus Corporation, Japan), with which the morphological parameters body length (BL) and crest width (CW) of primiparous *D*. *longicephala* were recorded. BL was defined as the distance from the upper edge of the compound eye to the starting point of the tail spine. CW was measured by drawing a line perpendicular to the body length axis and metering the distance from the ventral edge of the compound eye to the maximum extension of the crest (see supplementary material Fig. 2).

### Statistical analysis

Data from the control treatment and kairomone-exposed animals were separately analysed using SPSS (version 23.0, IBM, U.S.A.). All parameters were tested for normal distribution and homogeneity of variance. If assumptions for ANOVA were met, a one-way ANOVA followed by post hoc testing (Tukey HSD) was performed. Heteroscedastic data were analysed by performing a Welch test followed by a Tamhane T-2 post hoc test.

### Research animals

Experiments with animals were performed in accordance with relevant German guidelines and regulations.

## Supplementary information

Supplementary Information

## Data Availability

The data that support the findings of this study are available from the corresponding author upon reasonable request.
