# Supplementary material for: Plastic waste interferes with chemical communication in aquatic ecosystems
Source: Sci Rep. 2019 Apr 10;9:5889. doi: 10.1038/s41598-019-41677-1 (PMC6458178; doi:10.1038/s41598-019-41677-1)
Supplement: Supplementary file 1 — Supplementary Information [file 41598_2019_41677_MOESM1_ESM.pdf]

Plastic waste interferes with chemical communication in aquatic ecosystems

B. Trotter, A.F.R.M. Ramsperger, P. Raab, J. Haberstroh, C. Laforsch

Kairomone characterization:

Kairomones are not chemically defined compounds but they have an ecological definition: Kairomones are semiochemicals that bring benefit to the acceptor of the chemical signal while not being beneficial for the emitter. However, the nature of those chemicals is not known in most cases, since activity might be a result of several chemicals that act synergistically (see for example Pohnert et al[25]. So far the following characteristics have been described for kairomones of several predators:

- low molecular weight (<500 Da)[21,22]
- non-volatile, and anionic compound of medium polarity[21,22]
- extreme pH (0.8\pH\14.0) and temperature (-20°C-120°C) stability
- proteinase and peptidase resistant[21,22]
- hydroxyl groups are revealed as essential for biological activity of the kairomone [17,22]
- methyl groups and amine groups might be a substantial part of kairomones
- loses its activity under non-sterile conditions due to microbial degradation[21].

As the exact chemical structure of kairomones is not known in many cases, a direct measurement of kairomone concentration is not feasible. Since the expression of the morphological traits is following a dose-response curve, kairomone concentration is measured via the reaction norm of the phenotypically plastic traits.

Supplementary Results

Plastic exposition in kairomone-free water of the control (C), C+HDPE, C+PET treatments as well as the glass exposition C+GLASS had no effects on BL (Kruskal-Wallis, (χ^2 (3) = 5.329, p = 0.149) (Fig. 2D) or days until primiparity (Kruskal-Wallis, (χ^2 (3) = 3.081, p = 0.379) (Fig. 2F). An effect of plastic waste on the control animals was observed for the absolute crest width (CW) (one-way ANOVA, $F_{3,126} = 3.334$; $p < 0.05$) (Fig. 2E). Daphnids of the C+HDPE showed a slightly increased crest width (CW) compared to the control animals (Tukey HSD, p = 0.049).

Supplementary Discussion

Effects of leached additives

An increase in crest width was found in the C+HDPE treatment, which is absent of kairomones. Additives which may leach out of the plastics may evoke those effects. Yang et al.[S1] were able to show that different plastic products used for food packaging leached additives, which may have endocrine activity, in saline medium as well as in ethanol. Among those products were some made of the polymers HDPE and PET, both polymers found in our experimental setup. Of the two polymers HDPE only leached detectable additives in 61% of scenarios whereas PET leached in 100%. Yet, this leaching was measured after the polymers were exposed to ultraviolet radiation (wavelength 254nm) and incubated at a temperature of 37° C for a duration of 72h. As leaching is enhanced by UV radiation, as well as high temperature it is unlikely that leaching took place in our experimental, as there was no UV exposure, temperature was 17°C lower and experimental time was a third of the time used in the study by Yang et al.[41]. Furthermore, only a single morphological parameter is affected in our study. If leachates would stimulate the development of defensive structures, it is anticipated that every morphological parameter would be affected. In addition, as leaching of additives from PET was more distinct

in the study of Yang et al.[S1], we would have expected morphological changes rather in den C+PET daphnids compared to the C+HDPE daphnids were a slight crest induction was observed. Since the effect of additives was not the focus of our study we did not conduct any leachate analysis of the plastics and the surrounding medium. Hence, the discussion on the effects of possible leachates is highly speculative. However, that possible leachates from the used plastics did not affect *Daphnia* in our study is further supported, by the study of Lithner et al.[S2], where the effects on *D. magna* by leachates from various plastics were tested. Here 20 g of plastic were dispersed in 200 ml of deionized water and shaken for 24 h at a temperature of 20 ± 2°C, making conditions similar to our experiment. Yet, the leachates of HDPE did not show any effects on *Daphnia*.

Value of our study for future studies on inducible defenses

For our positive control, namely, the glass treatment, the following insights can be applied to past and future studies investigating kairomones. In our experiment, the I+GLASS daphnids were smaller in body length (BL) and crest width (CW) and reached primiparity earlier than the solely kairomone-exposed daphnids (I). These findings imply that glass also adsorbs kairomones to its surface, thereby reducing the perceivable kairomone concentration. Usually, authors state the ratio of predators per liter of medium in their induction experiments. With our results in mind, we suggest that the surface-to-volume ratio of the experimental container should also be reported to facilitate comparability among studies.

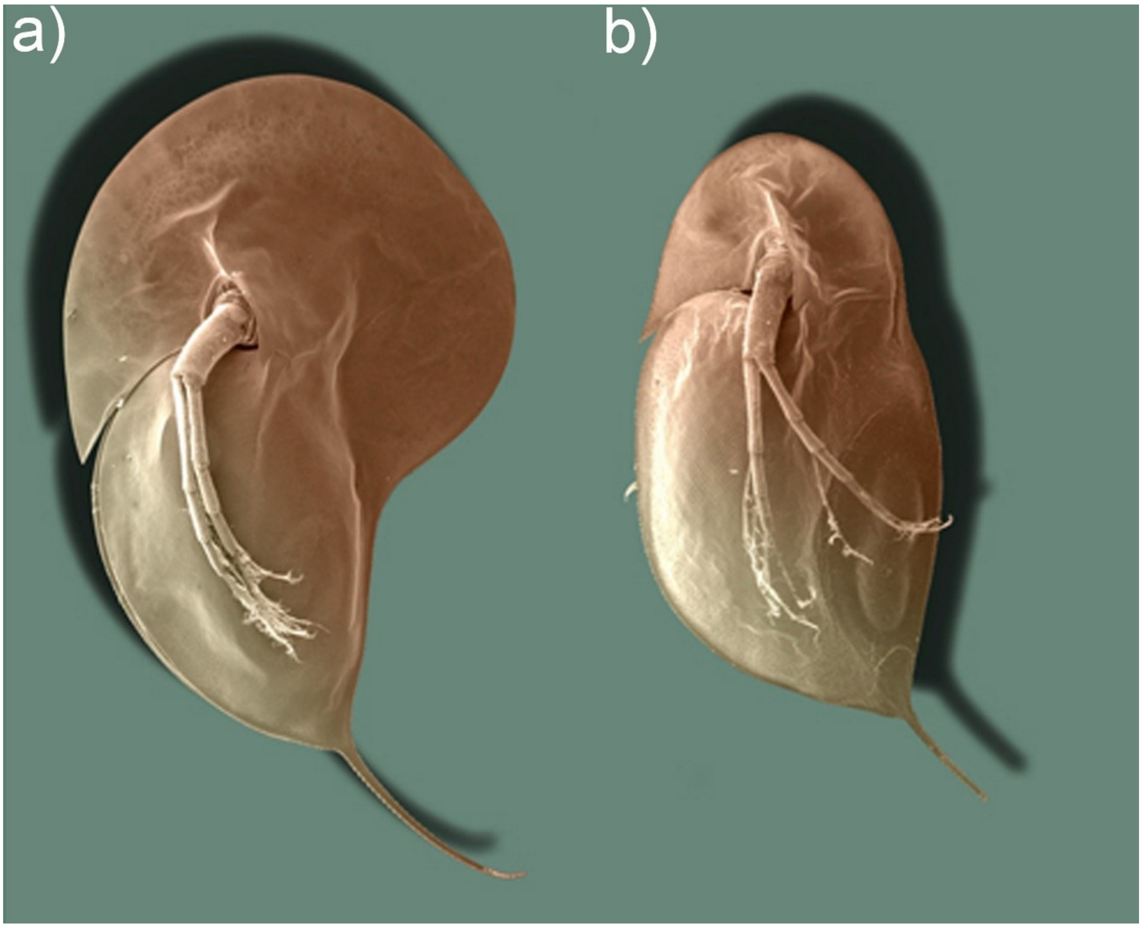

Fig. S 1: Comparison between two genetically identical individuals of *D. longicephala*. a) predator exposed ("induced") morph of *D. longicephala* with the prominent dorsal crest, which is formed in response to kairomones released by *Notonecta glauca,* and **b)** *D. longicephala* which has not experienced kairomones, therefore lacking the prominent dorsal crest. The expression of the morphological traits is following a dose-response curve. The higher the kairomone concentration is the higher is the reaction norm (crest width). Figure altered after Jeschke et al.[S3].

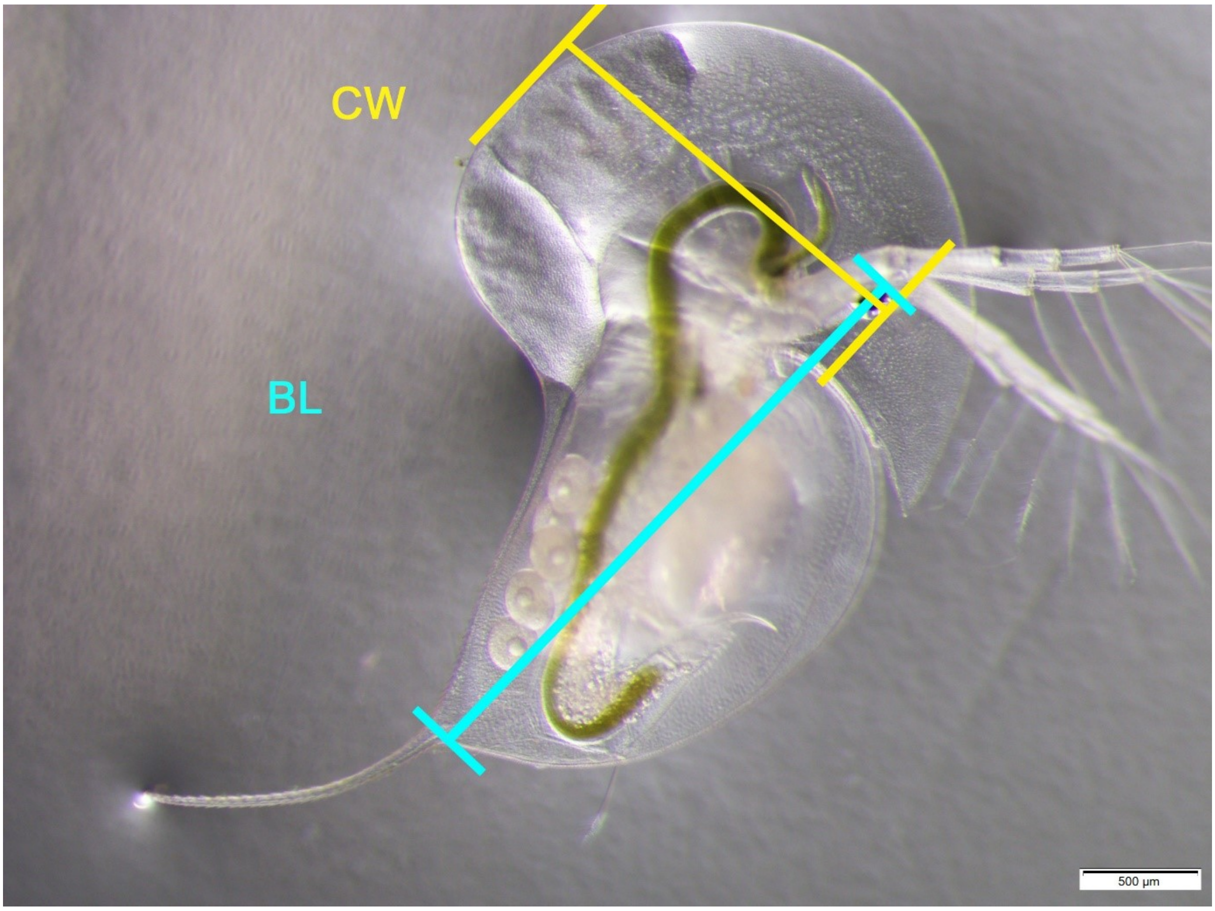

Fig. S 2: Induced *D. longicephala* and explanation of the measured morphological traits body length (BL) and crest width (CW).

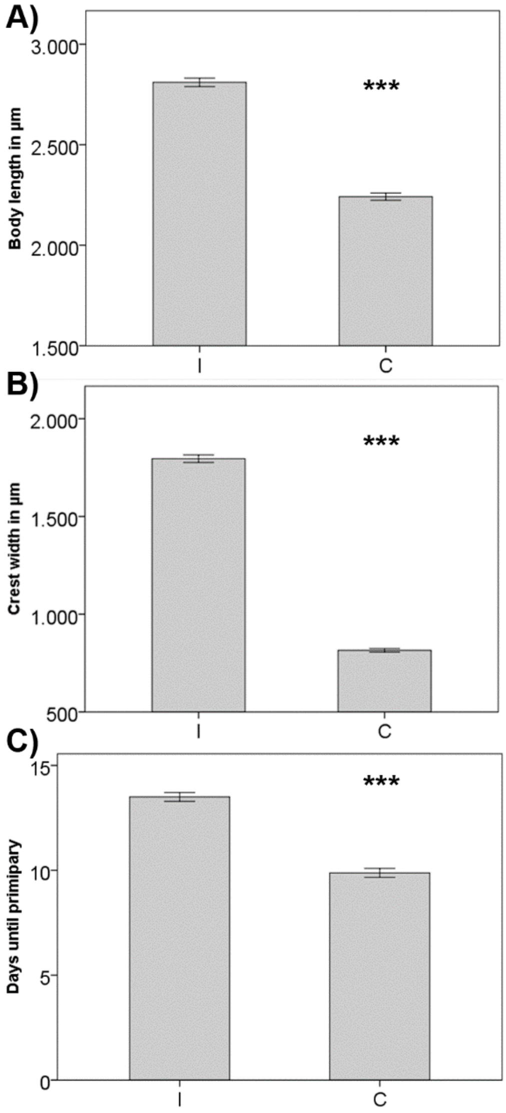

Fig. S 3: Effects of kairomone on morphological and life history parameters of *D. longicephala*: A) body length of daphnids exposed to kairomones compared to control daphnids lacking exposure to kairomones; **B)** crest width of daphnids exposed to kairomones compared to control daphnids lacking exposure to kairomones; **C)** duration in days until daphnids reach primiparity when exposed to kairomones compared to control daphnids lacking exposure to kairomones. Error bars indicate standard error of means (±1SE) Asterisks represent level of significance when treatments are compared with induction (I) or control (C) treatments: n.s. = $p \geq 0.05$; * = $p \leq 0.05$; *** = $p \leq 0.001$

Supplementary references

S1. Yang, C. Z., Yaniger, S. I., Jordan, V. C., Klein, D. J. & Bittner, G. D. Most plastic products release estrogenic chemicals: A potential health problem that can be solved. Environ. Health Perspect. 119, 989–996 (2011).

S2. Lithner, D., Damberg, J., Dave, G. & Larsson, Å. Leachates from plastic consumer products - Screening for toxicity with Daphnia magna. Chemosphere 74, 1195–1200 (2009).

S3. Jeschke, J. M., Laforsch, C. & Tollrian, R. in Encyclopedia of Ecology 189–194 (Elsevier, 2008).
